# Impaired cell–cell communication and axon guidance because of pulmonary hypoperfusion during postnatal alveolar development

**DOI:** 10.1186/s12931-023-02319-3

**Published:** 2023-01-11

**Authors:** Debao Li, Jing Wang, Yuan Fang, Yuqing Hu, Yingying Xiao, Qing Cui, Chuan Jiang, Sijuan Sun, Hao Chen, Lincai Ye, Qi Sun

**Affiliations:** 1 Department of Thoracic and Cardiovascular Surgery, Shanghai Children’s Medical Center, School of Medicine, Shanghai Jiao Tong University, 1678 Dongfang Road, Shanghai, 200127 China; 2 Department of Infectious Diseases, Shanghai Children’s Medical Center, School of Medicine, Shanghai Jiao Tong University, Shanghai, China; 3 Department of Plastic and Reconstructive Surgery, School of Medicine, Shanghai Ninth People’s Hospital, Shanghai Jiao Tong University, Shanghai, China; 4 Department of Cardiology, Shanghai Children’s Medical Center, School of Medicine, Shanghai Jiao Tong University, Shanghai, China; 5 Department of Pediatric Intensive Care Unit, Shanghai Children’s Medical Center, School of Medicine, Shanghai Jiao Tong University, Shanghai, China; 6 Institute of Pediatric Translational Medicine, Shanghai Children’s Medical Center, School of Medicine, Shanghai Jiao Tong University, Shanghai, China; 7 Shanghai Institute for Pediatric Congenital Heart Disease, Shanghai Children’s Medical Center, School of Medicine, Shanghai Jiao Tong University, 1678 Dongfang Road, Shanghai, 200127 China

**Keywords:** Alveolar dysplasia, Pulmonary hypoperfusion, COVID-19, Alveoli, Congenital heart diseases

## Abstract

**Background:**

Pulmonary hypoperfusion is common in children with congenital heart diseases (CHDs) or pulmonary hypertension (PH) and causes adult pulmonary dysplasia. Systematic reviews have shown that some children with CHDs or PH have mitigated clinical outcomes with COVID-19. Understanding the effects of pulmonary hypoperfusion on postnatal alveolar development may aid in the development of methods to improve the pulmonary function of children with CHDs or PH and improve their care during the COVID-19 pandemic, which is characterized by cytokine storm and persistent inflammation.

**Methods and results:**

We created a neonatal pulmonary hypoperfusion model through pulmonary artery banding (PAB) surgery at postnatal day 1 (P1). Alveolar dysplasia was confirmed by gross and histological examination at P21. Transcriptomic analysis of pulmonary tissues at P7(alveolar stage 2) and P14(alveolar stage 4) revealed that the postnatal alveolar development track had been changed due to pulmonary hypoperfusion. Under the condition of pulmonary hypoperfusion, the cell–cell communication and axon guidance, which both determine the final number of alveoli, were lost; instead, there was hyperactive cell cycle activity. The transcriptomic results were further confirmed by the examination of axon guidance and cell cycle markers. Because axon guidance controls inflammation and immune cell activation, the loss of axon guidance may explain the lack of severe COVID-19 cases among children with CHDs or PH accompanied by pulmonary hypoperfusion.

**Conclusions:**

This study suggested that promoting cell**–**cell communication or supplementation with guidance molecules may treat pulmonary hypoperfusion–induced alveolar dysplasia, and that COVID-19 is less likely to cause a cytokine storm in children with CHD or PH accompanied by pulmonary hypoperfusion.

**Graphical Abstract:**

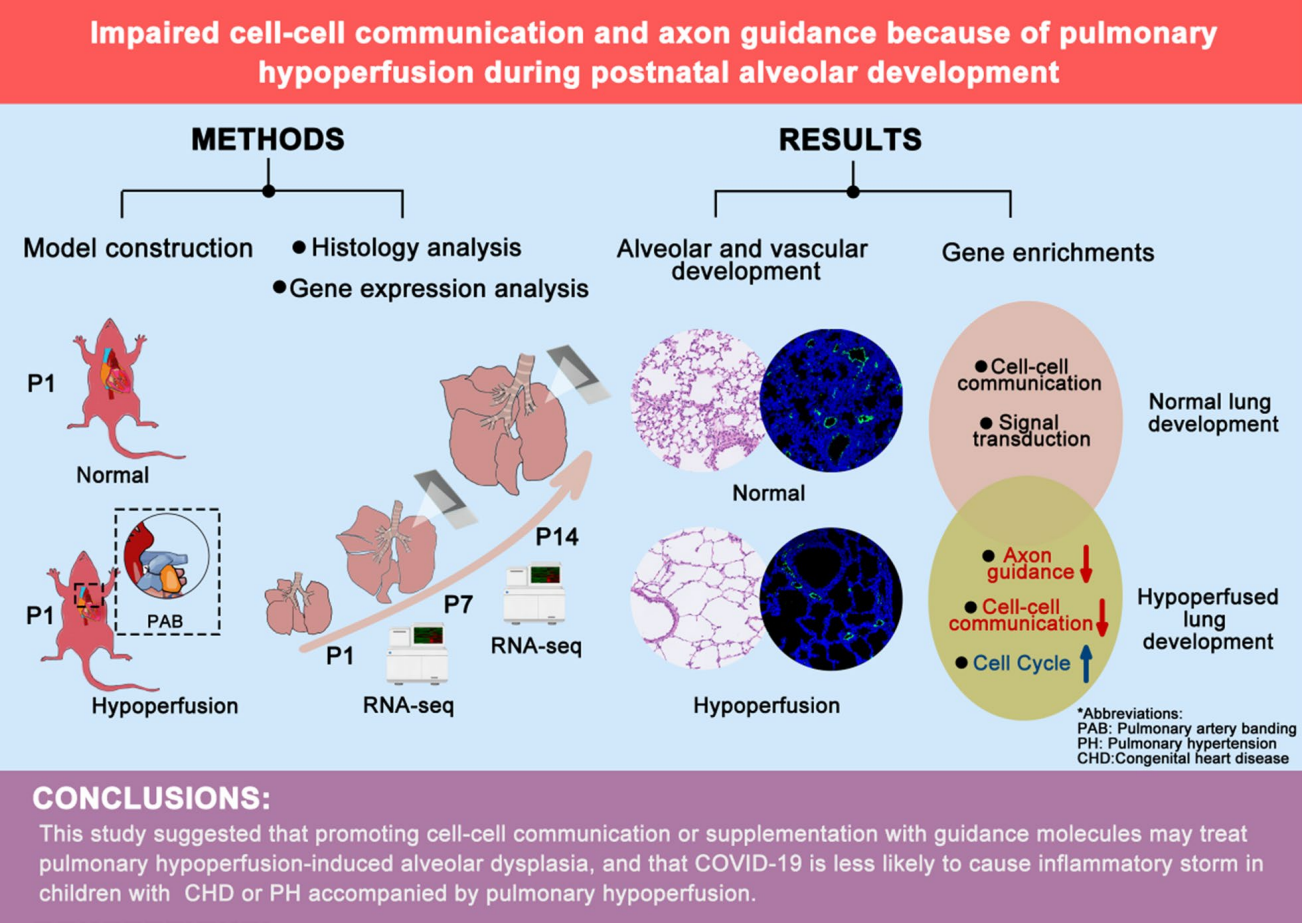

**Supplementary Information:**

The online version contains supplementary material available at 10.1186/s12931-023-02319-3.

## Clinical perspective

What’s new: The underlying mechanisms by which pulmonary hypoperfusion causes adult pulmonary dysplasia and affects postnatal alveolar development are revealed.

Clinical implications: Pulmonary hypoperfusion damages the cell-cell communication and axon guidance, suggesting that promoting cell-cell communication or supplementation with guidance molecules may treat pulmonary hypoperfusion-induced lung dysplasia. COVID-19 is less likely to cause an cytokine storm in the lungs of CHD children with pulmonary hypoperfusion due to the loss of axon guidance.

## Introduction

Pulmonary hypoperfusion is common in children with congenital heart diseases (CHDs), such as tetralogy of Fallot (TOF), severe pulmonary stenosis, pulmonary atresia, and palliative CHD surgery [1–3]. In addition, when the right ventricle fails due to chronic pulmonary hypertension (PH), patients develop pulmonary hypoperfusion [4, 5]. Previous studies demonstrated that children with pulmonary hypoperfusion are at risk of developing lung dysplasia as adults [6–8]. However, the underlying mechanisms by which pulmonary hypoperfusion causes lung dysplasia are unclear.

During the COVID-19 pandemic and the era of long COVID-19 syndrome, an increasing number of children with CHDs or PH have become infected with COVID-19 [9]. COVID-19 mainly damages the lungs, and a previous study established that COVID-19 causes a slowly unfolding, spatially limited alveolitis, in which alveolar macrophages containing COVID-19 and T cells form a positive feedback loop that drives persistent alveolar inflammation. This may explain long COVID-19 syndrome [10]. Whether and how pulmonary hypoperfusion is connected with COVID-19 are largely unknown. Understanding the underlying mechanisms may suggest strategies to alleviate the impact of COVID-19 in children with CHD or PH.

In humans, more than 90% of alveoli are formed after birth, especially between the ages of 0 and 7 years, and alveolar growth continues until adolescence [11, 12]. A rodent transcriptomic study revealed that postnatal alveolar development occurs in four stages: alveolar stage 1 (ALV1; postnatal days [P] 1–3), ALV2 (P4–7), ALV3 (P9–12), and ALV 4 (P12–18) [13]. In addition, rodent and human transcriptomics showed that the pathways associated with cell cycle and axon guidance were conserved between the postnatal alveolar development stages [13]. Cell cycle activation is required to achieve an adequate final number of alveoli, which is determined by the co-expansion of vascular endothelial cells and alveolar type (AT) 2 cells [14, 15]. Axon guidance acts in concert with other critical growth and morphogenetic factors to sculpt the architecture of the respiratory tree [16–18]. A recent study reported that AT1 cells function as a distinct signaling hub in the cell–cell communication that drives postnatal alveolar development [19]. It is unclear whether and how pulmonary hypoperfusion affects the aforementioned processes.

To fully understand how pulmonary hypoperfusion alters postnatal alveolar development, we constructed a neonatal rat pulmonary hypoperfusion model using pulmonary artery banding (PAB) at P1, as reported in our previous publications [20–22], and followed up the model at P21. Lung samples obtained at two time points (P7; ALV2 stage) and P14 (ALV4 stage) were selected for transcriptomic analysis to understand the developmental changes under the influence of pulmonary hypoperfusion. Based on transcriptomic data, cell cycle and axon guidance markers were tested further.

## Materials and methods

### Animals and PAB surgery

The pregnant rats (Xipu’er-bikai Experimental Animal Co, Ltd., Shanghai, China) were housed individually with free access to food and water, and maintained at a 12:12-h day:night cycle. The pups received PAB surgery or sham operation at P1, as previously described [9, 21, 22]. Briefly, neonatal rats were anesthetized using hypothermia on ice for 3 min and then placed on supine position on an ice bed to maintain anesthesia. A transectional sternal incision was performed at the third intercostal space to expose the chest cavity. The pericardium was pulled apart and both atria were separated to expose the aorta and the main pulmonary artery (PA). A 12–0 nylon suture was passed through the bottom of the PA and ligated using a 30-gauge padding needle. The needle was subsequently removed from the PA and a stenosis of the same diameter as the needle was created. The thoracic cavity was closed and the skin was sutured in layers. The pups were placed on a thermostatic warming plate for postoperative recovery and then placed back with their mothers after they recovered consciousness and could move freely. The sham pups received the same operation except for the banding step.

### Study design

A total of 50 pups were randomly divided into the sham and PAB groups. Lung tissues from sham and PAB groups were harvested at P7 and P14, called Sham_7, PAB_7, Sham_14, and PAB_14, respectively. At P7, all rats received echocardiography to confirm pulmonary hypoperfusion. At P7 and P14, five rats in each group and at each time point were used for RNA-seq, immunohistological analysis, Western blotting, and qRT-PCR. At P21, five rats in each group were used for morphological examinations and immunohistological analysis.

### Transthoracic echocardiography

To evaluate the pressure gradient and blood flow through the PA or left ventricular outflow tract, transthoracic echocardiography was performed by a single experienced echocardiologist. At P7, Rats were placed in a box with 5% isoflurane for 3–5 min to induce anesthesia and then put on a warming plate with a nasal cone containing 2.5% isoflurane to maintain anesthesia. A Vevo 2100 echocardiography system (Visual Sonics, Toronto, Ontario, Canada) and 25-MHz transducer (MS400 Micro Scan transducer; Visual Sonics) were used to perform the echocardiography. The blood flow patterns were obtained during pulsed Doppler modes from the long-axis view of the main PA and left ventricular outflow tract. Simultaneous measurements of the velocity–time integral (VTI), PA diameter, and the heart rate allowed the calculation of pulmonary blood flow using the following formula: ${pulmonary\,blood\,flow=(AOD \div 2)}^{2}\times \pi \times VTI\times HR$. The peak pressure gradient (PPG) was also recorded to demonstrate the presence of PA stenosis.

### Morphological examination

After the rats were euthanized, the rat thoracic cavity was opened to expose and remove the lungs. The lungs were washed with cold phosphate-buffered saline (PBS) to clear the blood from the lungs. An image of the gross lung morphology was obtained using a Leica M205 FA stereomicroscope. For histological and immunohistological analyses, the lungs were fixed and inflated by infusing 4% paraformaldehyde (pH 7.4) via the trachea (0.2 mL/10 g, 5 cmH_2_O) for 30 min in situ. Then, the lungs were removed, rinsed with PBS, and fixed at room temperature with 4% paraformaldehyde (pH 7.4) overnight. The tissues were dehydrated in gradient ethanol series, embedded in paraffin, and sliced into 5-µm sections. The sections were stained with hematoxylin and eosin (H&E) (Solarbio, Shanghai, China), according to the manufacturer’s instructions.

### Immunofluorescence

The paraffin-embedded lung sections were dewaxed and cleared with xylene, followed by hydration with gradient ethanol. Then, the sections were retrieved with sodium citrate solution (pH 8.0; Beyotime, Shanghai, China) for 20 min. The sections were blocked with blocking solution (7.5% goat serum and 0.5% Triton X-100 in PBS; Beyotime, Shanghai, China) for 1 h and incubated with primary rabbit (anti-CD31, Abcam, ab6994, 1:200) anti-Sema7a (Abcam, ab23578, 1:200), anti-RAGE (Abcam, ab21171, 1:200), and anti-Ki67 (Abcam, ab15580, 1:1000) antibodies overnight at 4 °C. The sections were washed twice with PBST (PBS with 1% tween) and once with PBS, and then incubated with secondary antibodies and DAPI mix for 1 h at room temperature. After washing for three times with PBS, the sections were covered with anti-florescence quencher and sealed with nail polish.

TUNEL staining was applied using the one-step TUNEL apoptosis assay kit according to the manufacturer's instructions. Briefly, the sections were dewaxed, rehydrated, and retrieved using antigen. Then, the sections were incubated with 20 μg/mL of protein K for 30 min at room temperature. After washing with PBS, the sections were incubated with 50 µL of TUNEL detection solution (5 µL of TdT enzyme and 45 µL of fluorescein labeling solution) at 37 °C for 1 h.

### Alveolarization and vascularization assessment

Lung alveolarization was evaluated using mean linear intercept (Lm, estimating alveolar size). The measurement of Lm was performed as previously described [23, 24]. Briefly, a horizontal line and a vertical line were drawn on an H&E image so that the intersection point of the lines coincided with the center point of a terminal bronchiole. The total length of the two lines and number of the times that the two lines intersect with the alveoli were recorded. Lm was defined as Lm $= (total\,lines\,length \div total\,intersected\,times)$ in μm.

Lung vascularization was determined using the quantification of blood vessel density, as demonstrated by endothelial marker CD31 intensity [23, 24]. Images were obtained from ten random, non-overlapping (n = 5/group) 20 × fields from lung apex to base and processed using Image J software (www.rsb.info.nih.gov/ij).

### Total RNA preparation and real-time quantitative analysis

The total mRNA was extracted and purified using a PureLink RNA Micro Scale Kit (Catalog no. 12183016; Life Technologies, Carlsbad, CA, USA), and then reversed using a PrimeScript reagent kit (Takara Bio, Kusatsu, Japan). Then, qPCR was performed using SYBR Green Power Premix Kits (Applied Biosystems, Foster City, CA, USA) with a 7900 Fast Real-Time PCR System (Applied Biosystems), according to the manufacturer’s instructions, under the following conditions: 1 cycle at 95 °C for 10 s, followed by 40 cycles of 95 °C for 15 s and 60 °C for 60 s. The primers were obtained from Generay Biotech Co., Ltd. (Shanghai, China). The relative fold change was calculated using the ΔΔCT method.

### RNA sequencing and analyses

A total of 1 μg of RNA per sample was used as input material for RNA sample preparation. Sequencing libraries were generated using the NEBNext® UltraTM RNA Library Prep Kit for Illumina® (NEB, USA) following the manufacturer’s instructions. Library quality was assessed using an Agilent Bioanalyzer 2100 system.

Clustering was generated with TruSeq PE Cluster Kit v3-cBot-HS (Illumina) on a cBot Cluster Generation System and then sequenced on an Illumina Novaseq platform according to the manufacturer’s instructions.

Clean data (clean reads) were obtained by removing reads containing adapters, reads containing poly-N, and low-quality reads from the raw data. HISAT2 v2.5.0 was used to align the sequence reads to the rat reference genome (assembly rnor_6.0) and featureCounts v1.5.0-p3 was used to count the read numbers mapped to each gene. Expression values were determined by calculating the number of fragments per kilobase of transcript sequence per million base pairs sequenced (FPKM).

Differential expression analysis was performed using the DESeq2 R package (1.20.0). The resulting p-values were adjusted using Benjamini and Hochberg’s approach for controlling the false discovery rate. Genes with an adjusted p-value < 0.05 determined by DESeq2 were considered differentially expressed.

Gene ontology (GO; http://www.geneontology.org) and Kyoto encyclopedia of genes and genomes (KEGG; http://www.genome.jp/kegg/) pathway enrichment analyses were performed using the cluster profile R package.

### Western blotting

Proteins were extracted using RIPA Lysis Buffer according to the manufacturer’s instruction, separated on 10% SDS polyacrylamide gels, and transferred onto polyvinylidene fluoride membranes (Merck, Millipore, Billerica, MA, USA). Then, the membranes were blocked with 5% non-fat milk in Tris-buffered saline with Tween 20 (TBST) for 1 h at room temperature and incubated with primary antibodies overnight at 4 °C. After washing thrice with TBST, the membranes were incubated with secondary antibodies for 1 h at room temperature and the proteins were detected using the Bio-Rad ChemiDoc™ Imaging Systems (Bio-Rad, Hercules, CA, USA).

### Statistical analysis

Continuous data are expressed as mean ± standard deviation. Differences were tested using one-way ANOVA test and Student Newman Keuls (SNK) post hoc test was applied. Categorical variables were compared using the Wilcoxon test. *p*-values < 0.05 were considered statistically significant. Statistical analyses were performed using SAS software (version 9.4; SAS Institute Inc., Cary, NC, USA).

## Results

### Construction of neonatal pulmonary hypoperfusion model

**Fig. 1:**
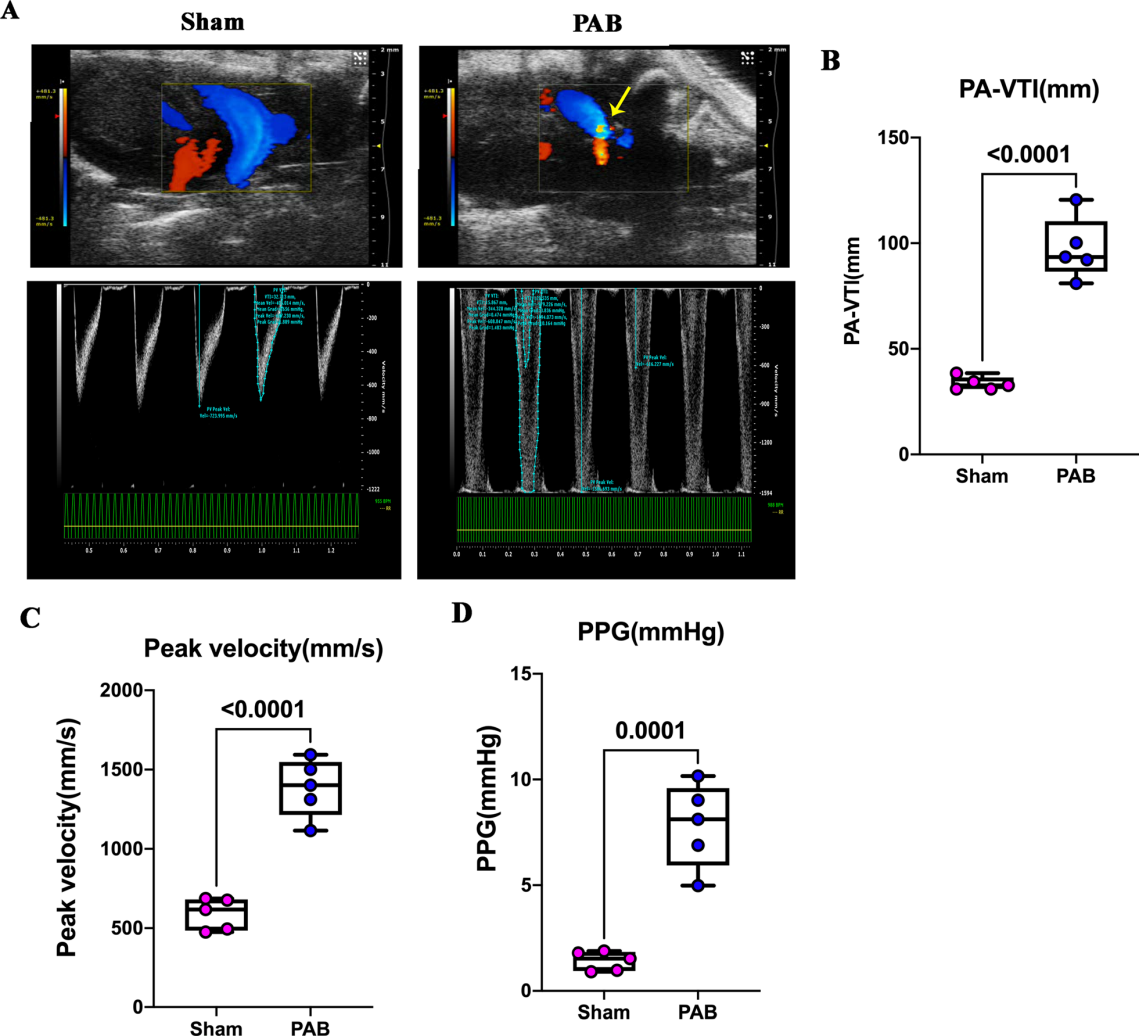
Construction of neonatal pulmonary hypoperfusion model by neonatal pulmonary artery banding (PAB). **A** Representative transthoracic echocardiography demonstrating the long-axis view of the main pulmonary artery (PA). The yellow arrow represents the narrow, irregular, and colorful blood flow signal, indicating a high-speed blood flow with a significant stenosis area. **B** Quantification of PA-VTI. **C** Quantification of peak-velocity. **D** Quantification of mean pressure gradient (PPG) across the PA

We created a neonatal pulmonary hypoperfusion model by performing PAB surgery on P1 rats, as reported previously [9, 21, 22]. The color Doppler ultrasound showed an irregular colorful blood flow signal accompanied with a significant narrowing in the PAB group (Fig. 1A, arrow), indicating a high-speed turbulent flow at the banding site due to stenosis. Quantification data showed that PA-VTI, peak velocity, and PPG in the PAB group increased by 4.9-, 2.5-, and 6.9-folds compared to those in the sham group, respectively (Fig. 1A–D). These results confirmed the successful creation of PA stenosis.

### Verification of neonatal pulmonary hypoperfusion model

**Fig. 2:**
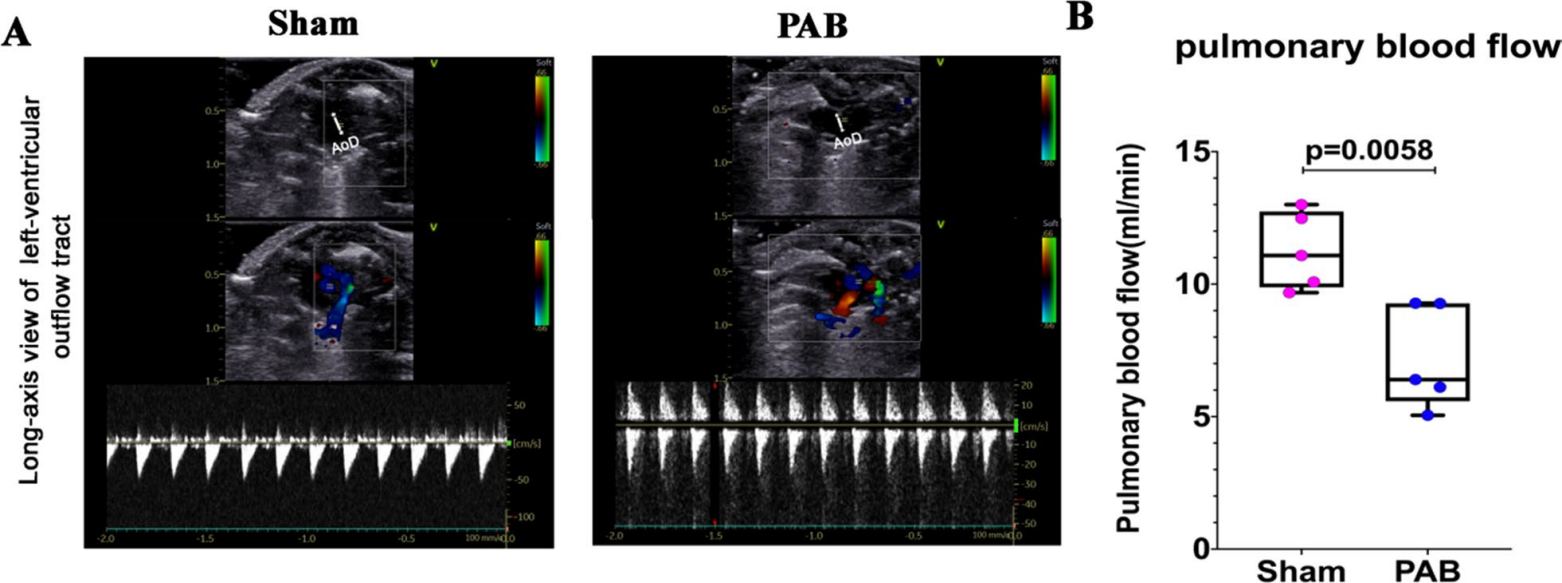
Validation of neonatal pulmonary hypoperfusion in PAB rats at postnatal day 7. **A** Representative transthoracic echocardiography demonstrating the long-axis view of the left ventricular outflow tract. **B** Quantification of the pulmonary blood flow in sham and PAB groups.

We calculated the pulmonary perfusion through the left ventricular outflow tract (Fig. 2A), and found that the pulmonary blood flow was significantly lower in the PAB group than in the sham group (Fig. 2B) at P7. These results suggested that the neonatal pulmonary hypoperfusion model was successfully created.

### Pulmonary hypoperfusion caused alveolar dysplasia

**Fig. 3:**
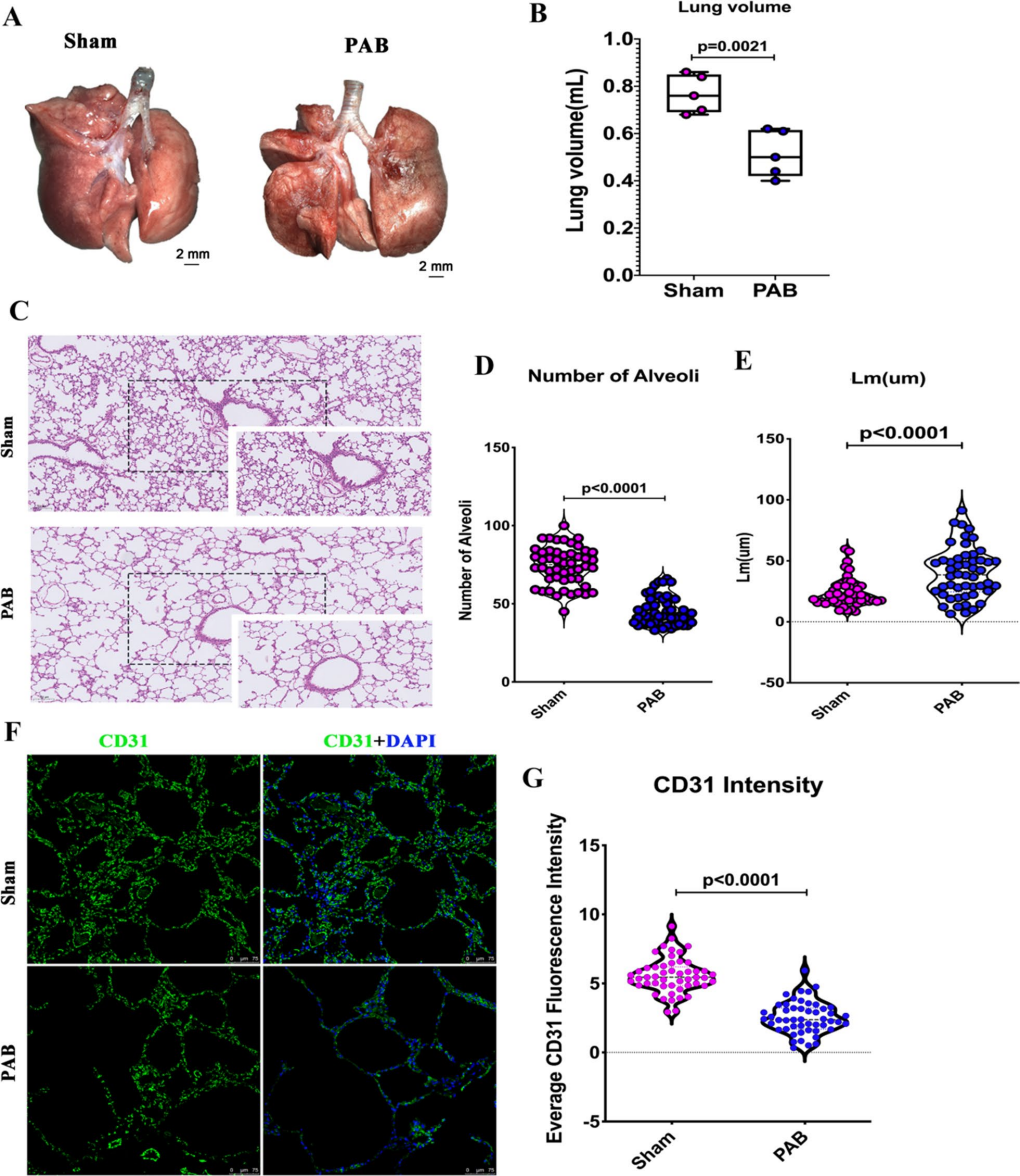
Pulmonary hypoperfusion caused alveolar dysplasia at postnatal day 21. **A** Representative gross image of the lungs. **B** Quantification of lung volume. **C** Representative CD31 immunostaining, indicating blood vessels in the lungs. **D** Quantification of CD31 intensity in sham and PAB groups. **E** Representative alveoli in sham and PAB groups. **F** Quantification of the number of alveoli per field in the sham and PAB groups. N = 50 fields from five samples. **G** Quantification of Lm in sham and PAB groups

At P21, smaller lungs and lung volume were observed in the PAB group (Fig. 3A, B). At the tissue level, there was a significant decrease in the number of alveoli and a significant increase in Lm in the PAB compared to the sham groups (Fig. 3C–E), suggesting impaired alveolarization due to PAB. Furthermore, a lower blood vessel density was also observed in the PAB than in the sham groups (Fig. 3F, G), indicating impaired vascularization due to PAB. These results confirmed that pulmonary hypoperfusion leads to alveolar dysplasia [9].

### Transcriptomics of postnatal alveolar development because of pulmonary hypoperfusion

**Fig. 4:**
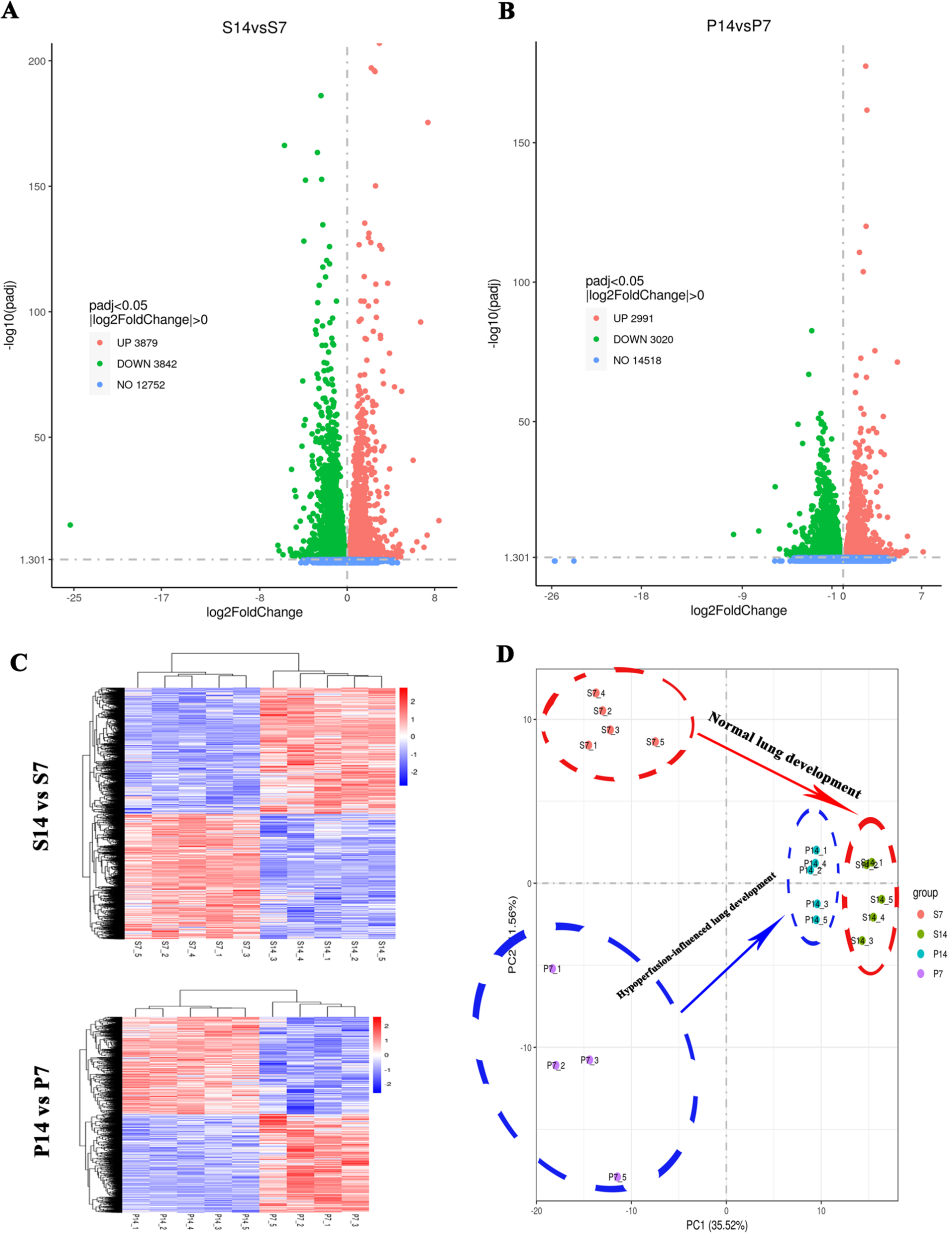
Pulmonary hypoperfusion changed the transcriptomics of postnatal alveolar development. **A** Volcano map of differentially expressed genes (DEGs) of postnatal alveolar development in the normal condition (Sham_14 [S14] vs. Sham_7 [S7]). **B** Volcano map of DEGs of postnatal alveolar development under the influence of pulmonary hypoperfusion (PAB_14 [P14] vs. PAB_7 [P7]). **C** Heat map for cluster analysis of DEGs. **D** PCA plots analysis of DEGs, indicating changed developmental track because of pulmonary hypoperfusion

To investigate how pulmonary hypoperfusion affects the gene expression during the AVL stage, we obtained lung tissues at P7 and P14 to perform RNA-seq analysis (n = 5, except for the P7 group, which contained four rats because one died before harvesting). As shown in the volcano map in Fig. 4A, during normal postnatal alveolar development, there were 7721 DEGs between the Sham_14 and Sham_7 groups, among which 3879 were upregulated and 3842 were downregulated. Under the influence of pulmonary hypoperfusion, this number decreased to 6011, of which 2991 were upregulated and 3020 were downregulated (Fig. 4B). The heat map revealed a high similarity between samples within the same group but apparent difference between samples from different groups (Fig. 4C), suggesting a high biological reproducibility of PAB surgery. The principal component analysis (PCA) plot further confirmed the high reproducibility of PAB surgery, and showed a different postnatal developmental track between normal and pulmonary hypoperfusion-influenced lungs (Fig. 4D). The differences between the PAB and sham groups were more significant at P7 than at P14 (Fig. 4D). These results demonstrated that the postnatal alveolar developmental track is changed by pulmonary hypoperfusion.

### Biological processes (BP), cellular components (CC), and molecular functions (MF) of postnatal alveolar development changed by pulmonary hypoperfusion as indicated by GO analysis

We performed GO analysis on the DEGs to identify the changes in BP, CC, and MF during postnatal alveolar development due to pulmonary hypoperfusion.

**Fig. 5:**
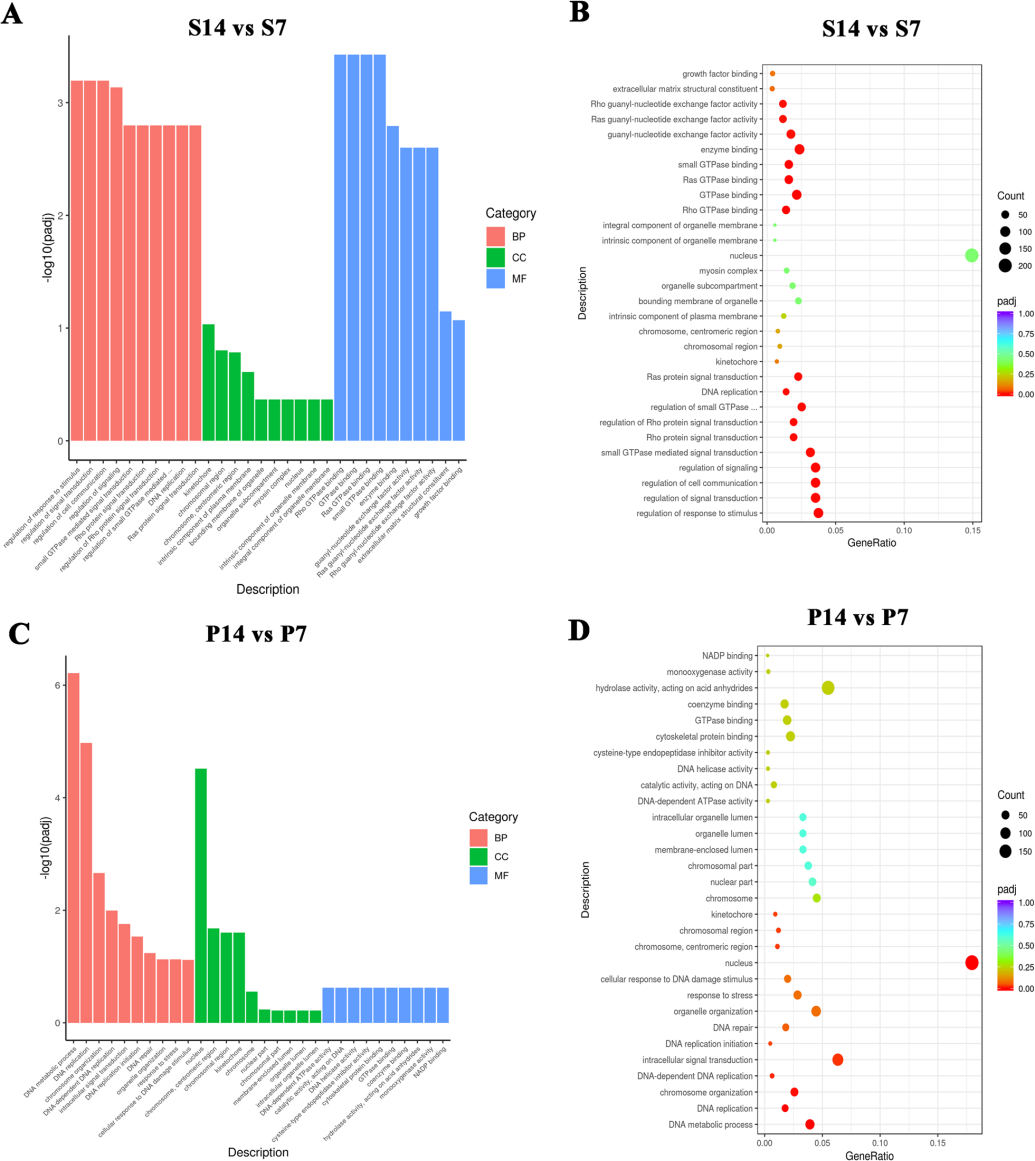
Cell–cell communication signal transduction during normal postnatal alveolar development were partly changed by cell cycle activity because of pulmonary hypoperfusion, as indicated by the GO analysis. **A** Based on the results of the GO enrichment analysis for normal postnatal alveolar development (S14 vs. S7), the most significant 10 terms are displayed. The abscissa is the GO term, and the ordinate is the significance level of GO term enrichment. The higher the value, the more significant the result. The different colors represent three different GO subclasses: biological process (BP), cellular component (CC), and molecular function (MF). **B** Based on the results of the GO enrichment analysis of normal postnatal alveolar development (S14 vs. S7), we selected the most significant 30 terms to draw scatter plots for display. The abscissa is the ratio of the number of DEGs on the GO term to the total number of DEGs, the ordinate is the GO Term, the size of the dots represents the number of genes annotated to the GO term, and the color from red to purple represents the significance level of GO term enrichment. The most enriched GO terms were associated with cell–cell communication signal transduction. **C** Based on the results of the GO enrichment analysis of pulmonary hypoperfusion influenced-postnatal alveolar development (P14 vs. P7), the most significant 10 terms are displayed. The abscissa is the GO term and the ordinate is the significance level of GO term enrichment. The higher the value, the more significant the result. The different colors represent three different GO subclasses: BP, CC, and MF. **B** Based on the results of the GO enrichment analysis of pulmonary hypoperfusion influenced-postnatal alveolar development (P14 vs. P7), we selected the most significant 30 terms to draw scatter plots for display. The abscissa is the ratio of the number of DEGs on the GO term to the total number of DEGs, the ordinate is GO Term, the size of the dots represents the number of genes annotated to the GO Term, and the color from red to purple represents the significance level of GO term enrichment. The most enriched GO terms were associated with cell cycle activity

During normal postnatal alveolar development, the top 10 most enriched GO terms of BP were regulation of response to stimulus, regulation of signal transduction, regulation of cell communication, regulation of signaling, small GTPase-mediated signal transduction, Rho protein signal transduction, regulation of Rho protein signal transduction, regulation of small GTPase-mediated signal transduction, DNA replication, and ras protein signal transduction (Fig. 5A, B). The top 10 most enriched GO terms of CC were kinetochore, chromosomal region, chromosome, centromeric region, intrinsic component of plasma membrane, bounding membrane of organelle, organelle subcompartment, myosin complex, nucleus, intrinsic component of organelle membrane, and integral component of organelle membrane (Fig. 5A, B). The top 10 most enriched GO terms of MF were Rho GTPase binding, GTPase binding, Ras GTPase binding, small GTPase binding, enzyme binding, guanyl-nucleotide exchange factor activity, Ras guanyl-nucleotide exchange factor activity, Rho guanyl-nucleotide exchange factor activity, extracellular matrix structural constituent, and growth factor binding (Fig. 5A, B). These results highlight the critical role of cell–cell communication and signal transduction during normal postnatal alveolar development, which is consistent with a previous publication [20].

Under the influence of pulmonary hypoperfusion, the top 10 most enriched GO terms of BP were DNA metabolic process, DNA replication, chromosome organization, DNA-dependent DNA replication, intracellular signal transduction, DNA replication initiation, DNA repair, organelle organization, response to stress, and cellular response to DNA damage stimulus (Fig. 5C, D). The top 10 most enriched GO terms of CC were nucleus, chromosome and centromeric region, chromosomal region, kinetochore, chromosome, nuclear part, chromosomal part, membrane-enclosed lumen, organelle lumen, and intracellular organelle lumen (Fig. 5C, D). The top 10 most enriched GO terms of MF were DNA-dependent ATPase activity, catalytic activity acting on DNA, DNA helicase activity, cysteine-type endopeptidase inhibitor activity, cytoskeletal protein binding, GTPase binding, coenzyme binding, hydrolase activity, acting on acid anhydrides, monooxygenase activity, and NADP binding (Fig. 5C, D). These results suggest a significant increase in cell cycle activity during pulmonary hypoperfusion-influenced postnatal alveolar development.

The aforementioned results suggest that cell–cell communication and signaling transduction during normal postnatal alveolar development were partly switched to cell cycle activity because of pulmonary hypoperfusion.

### Pathways of postnatal alveolar developmental trajectory were changed by pulmonary hypoperfusion

**Fig. 6:**
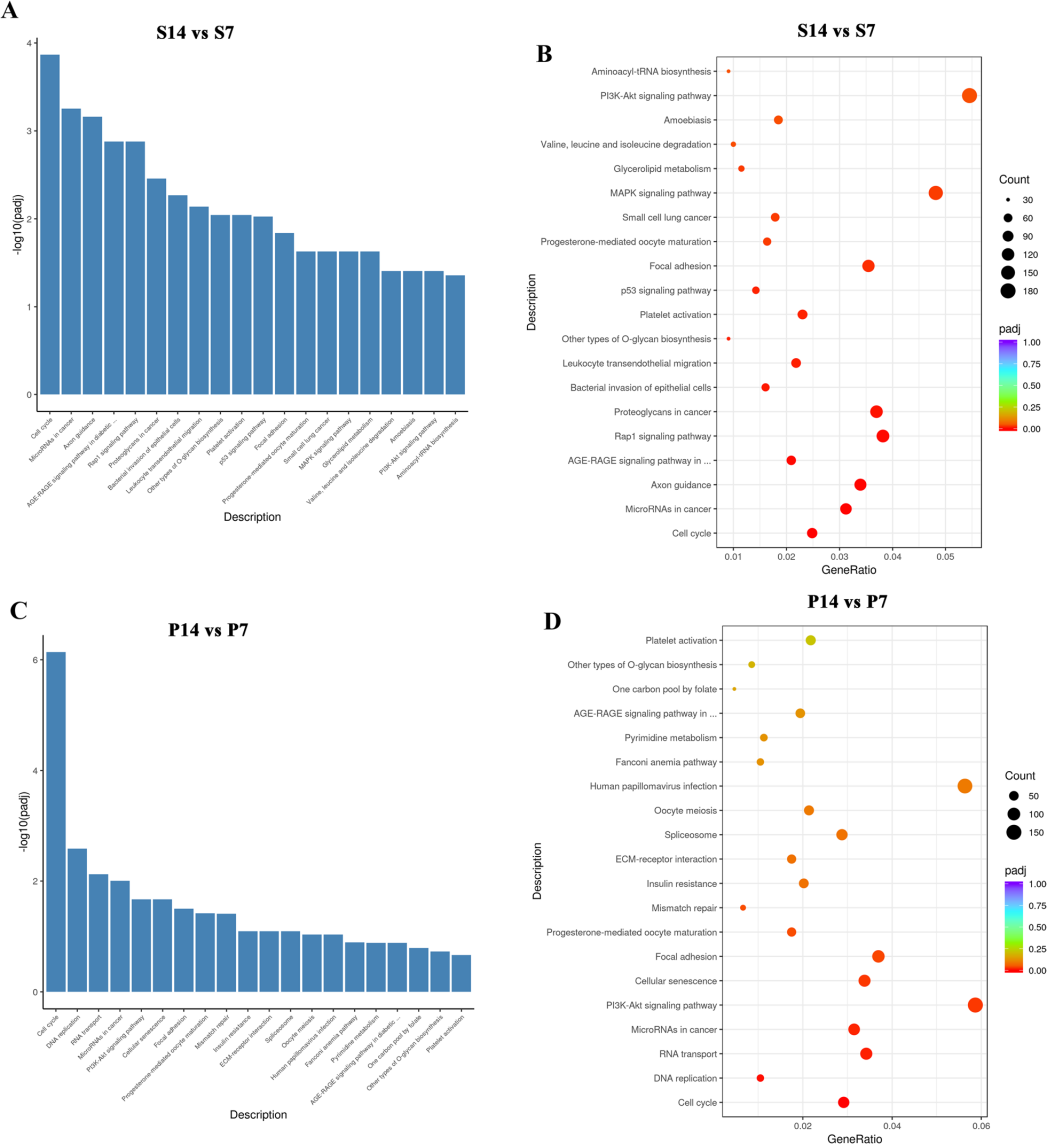
KEGG pathway analysis of the DEGs during normal postnatal alveolar development and postnatal alveolar development under the influence of pulmonary hypoperfusion. **A** KEGG pathways of normal alveolar development. The most significant 20 KEGG pathways from the KEGG enrichment results are displayed. The abscissa is the KEGG pathway and the ordinate is the significance level of pathway enrichment. The higher the value, the greater the significance. **B** The KEGG pathways of normal alveolar development. Based on the KEGG enrichment results, the most significant 20 KEGG pathways were selected for the scatter plots. The abscissa is the ratio of the number of DEGs on the KEGG pathway to the total number of DEGs, the ordinate is the KEGG pathway, the size of the dots represents the number of genes annotated to the KEGG pathway, and the color from red to purple represents the significance level of KEGG pathway enrichment. **C** The KEGG pathway of p ostnatal alveolar development under the influence of pulmonary hypoperfusion. The most significant 20 KEGG pathways from the KEGG enrichment results are displayed. The abscissa is the KEGG pathway and the ordinate is the significance level of pathway enrichment. The higher the value, the greater the significance. **D** The KEGG pathways of postnatal alveolar development under the influence of pulmonary hypoperfusion. Based on the KEGG enrichment results, the most significant 20 KEGG pathways were selected for the scatter plots. The abscissa is the ratio of the number of DEGs on the KEGG pathway to the total number of DEGs, the ordinate is the KEGG pathway, the size of the dots represents the number of genes annotated to the KEGG pathway, and the color from red to purple represents the significance level of KEGG pathway enrichment. Axon guidance is not included in the list of enriched KEGG terms, and the enriched score of cell cycle terms is much higher than those of other enriched terms

KEGG pathway analysis was used to identify the pathways regulating alveolar development that were affected by pulmonary hypoperfusion. The results indicated that the top 20 enriched pathways during normal alveolar development were cell cycle, microRNAs in cancer, axon guidance, AGE-RAGE signaling pathway in diabetic complications, rap1 signaling pathway, proteoglycans in cancer, bacterial invasion of epithelial cells, leukocyte transendothelial migration, other types of O-glycan biosynthesis, platelet activation, p53 signaling pathway, focal adhesion, progesterone-mediated oocyte maturation, small cell lung cancer, MAPK signaling pathway, glycerolipid metabolism, valine, leucine and isoleucine degradation, amoebiasis, PI3K-Akt signaling pathway, and aminoacyl-tRNA biosynthesis (Fig. 6A, B). These results suggest that cell cycle and axon guidance are the primary pathways that regulate normal postnatal alveolar development, consistent with a previous publication [15].

Under the influence of pulmonary hypoperfusion, the top 20 enriched pathways were cell cycle, DNA replication, RNA transport, microRNAs in cancer, PI3K-Akt signaling pathway, cellular senescence, focal adhesion, progesterone-mediated oocyte maturation, mismatch repair, insulin resistance, ECM-receptor interaction, spliceosome, oocyte meiosis, human papillomavirus infection, Fanconi anemia pathway, pyrimidine metabolism, AGE-RAGE signaling pathway in diabetic complications, one carbon pool by folate, other types of O-glycan biosynthesis, and platelet activation (Fig. 6C, D). Notably, the − log10 (padj) of cell cycle was 7.27 and 3.59 in the PAB and sham groups, respectively, and axon guidance was not included in the list of enriched terms (Fig. 6A, C). These results suggest that pulmonary hypoperfusion led to overactivation of the cell cycle and absent axon guidance during postnatal alveolar development.

### Verification of RNA-seq results by examination of cell cycle and axon guidance markers

**Fig. 7:**
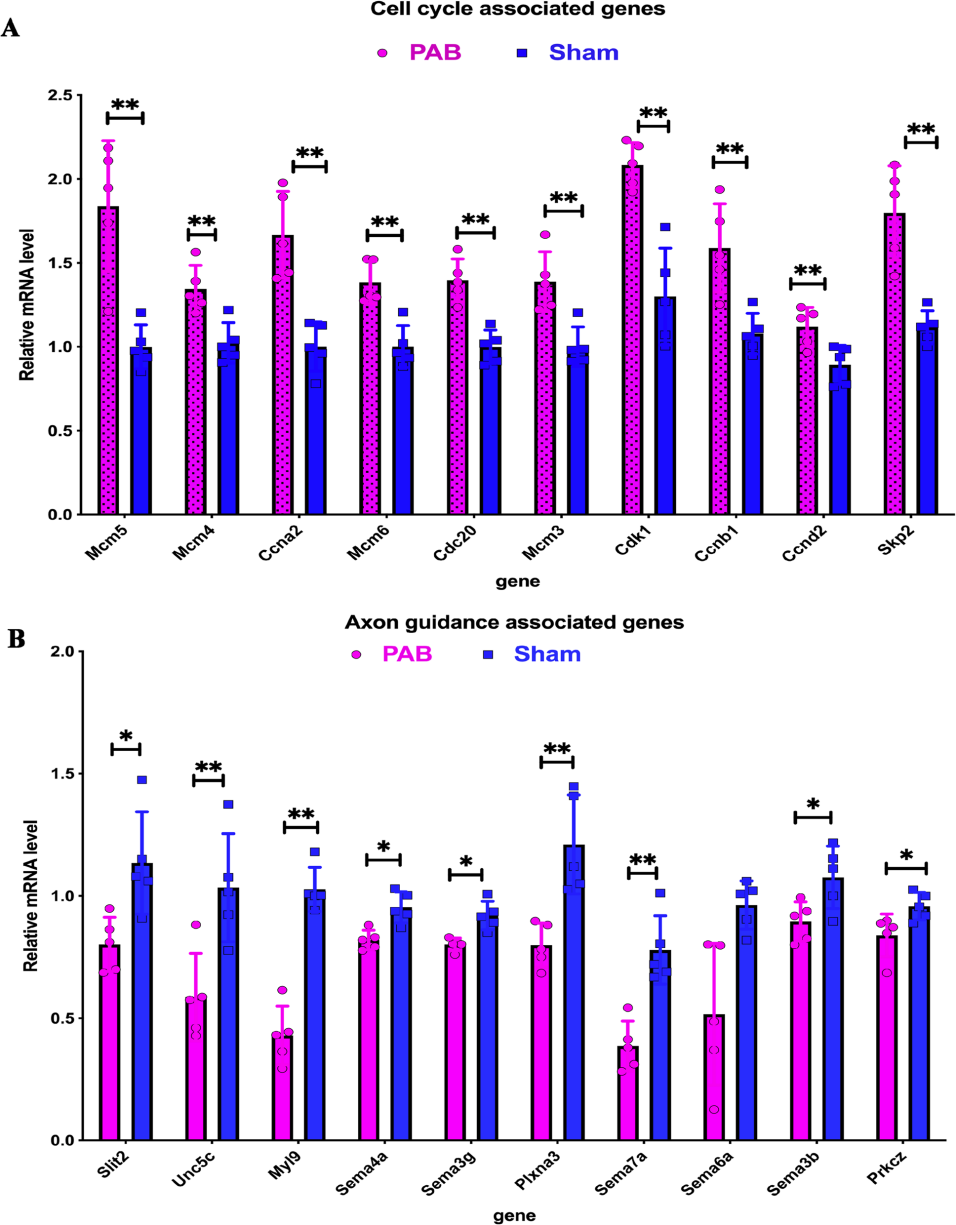
Verification of RNA-seq results by qRT-PCR at postnatal day 14. **A** The mRNA level of 10 randomly selected cell cycle-associated genes. These genes were upregulated in the PAB group. **B** The mRNA level of 10 randomly selected axon guidance-associated genes. These genes were downregulated in the PAB group.*p < 0.05,**p < 0.01, vs Sham, Student *t -test*

To confirm the RNA-seq results, 10 randomly selected cell cycle and axon guidance associated genes were verified by qRT-PCR (Fig. 7A, B). The results showed that the expression levels of cell cycle-associated genes were significantly higher (Fig. 7A), while the expression levels of axon guidance-associated genes were significantly lower (Fig. 7B), in the PAB group compared to the sham group. These results suggest that cell cycle was promoted and axon guidance was inhibited during postnatal alveolar development due to pulmonary hypoperfusion.

**Fig. 8:**
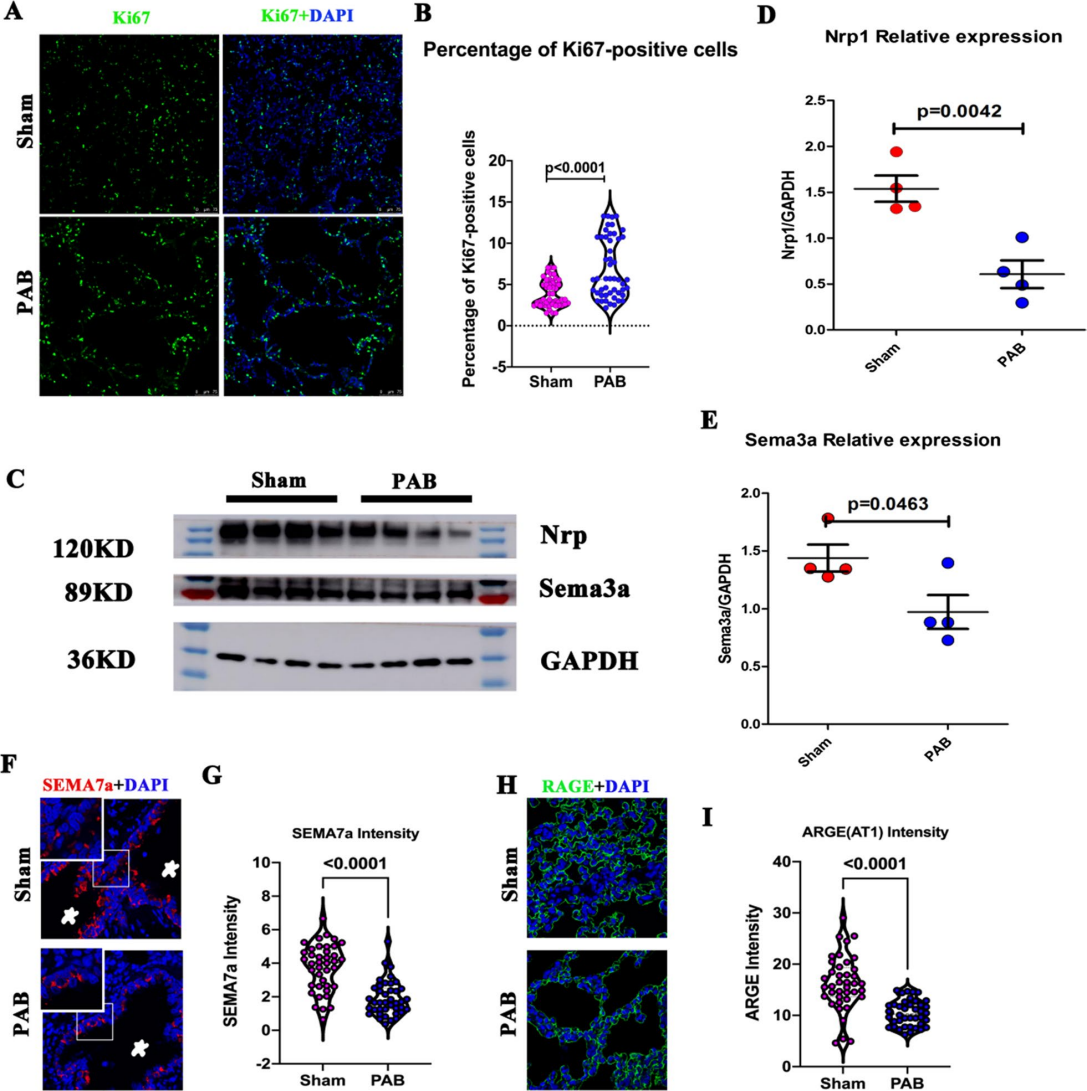
Verification of RNA-seq results by examination of the markers of cell cycle and axon guidance on postnatal day 14. **A** Representative Ki67-positive cells in the sham and PAB groups. Ki67 (green); DAPI (blue). **B** Quantification of Ki67-positive cells in the sham and PAB groups. **C** Representative blots of SMEAD3 and Nrp1 in the sham and PAB groups. **D** Quantification of Nrp1 relative blot density. **E** Quantification of SMEAD3 relative blot density. **F** Representative SEMA7a staining in the sham and PAB groups. SMEAD7 (Red); DAPI (blue). Stars indicate bronchioles. **G** Quantification of SEMA7a intensity. **H** Representative AT1 staining in the sham and PAB groups. RAGE (Green, a marker of AT1); DAPI (blue). **I** Quantification of AT1 intensity

Ki67, a cell cycle marker, was selected to confirm the RNA-seq results. As shown in Fig. 8A and B, the proportion of Ki67-positve cells was significantly increased in PAB group than in the sham group, indicating over-activated cell cycle because of pulmonary hypoperfusion. In contrast, the axon guidance markers SEMA3A and Nrp1 were lower in the PAB group than in the sham group (Fig. 8C–E), indicating inhibition of axon guidance caused by pulmonary hypoperfusion. To visualize semaphorin distribution and axon trajectories, we performed SEMA7a immunofluorescence staining, which showed that SEMA7a was located at bronchioles and downregulated under pulmonary hypoperfusion conditions (Fig. 8F, G). To assess cell**–**cell communication, we determined the expression of AT1 cells, a hub of cell**–**cell communication for postnatal alveolar development [19]. The results showed downregulation of AT1 cells under pulmonary hypoperfusion conditions (Fig. 8H, I).

**Fig. 9:**
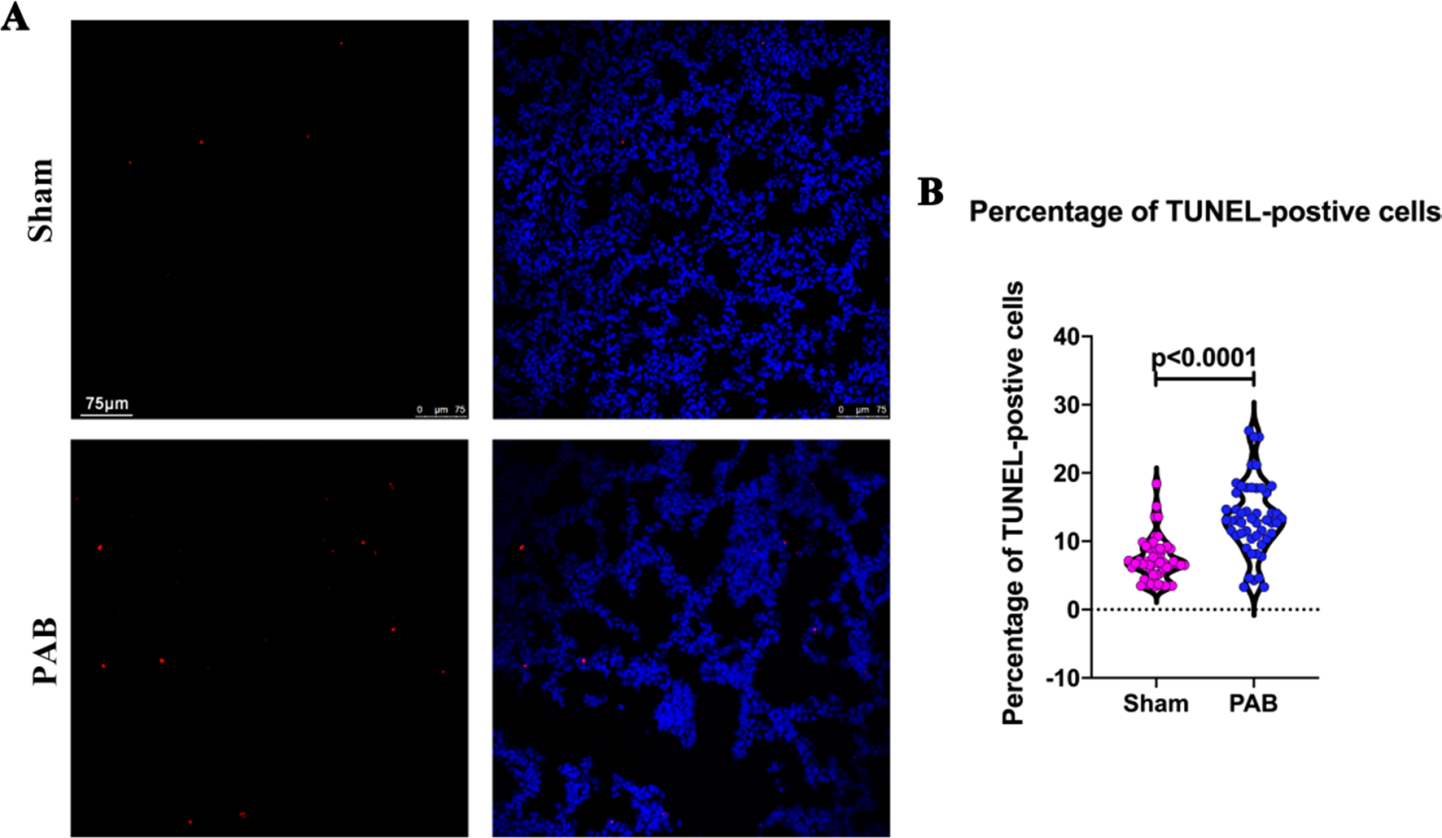
Pulmonary hypoperfusion increased apoptosis in postnatal lungs. **A** Representative TUNEL-positive cells in the sham and PAB groups. TUNEL (red); DAPI (blue). **B** Quantification of TUNEL-positive cells in the sham and PAB groups

Furthermore, we observed a significantly higher percentage of TUNEL-positive cells in the PAB group than in the sham group (Fig. 9A, B), suggesting higher apoptosis in the PAB group than the sham group because of pulmonary hypoperfusion.

## Discussion

Although it is well-known that pulmonary hypoperfusion impairs postnatal alveolar development and predisposes patients to adult lung dysplasia, the underlying mechanism is not clear. Pulmonary hypoperfusion patients demonstrate exercise intolerance and impaired quality of life. Currently, there are no effective strategies to prevent pulmonary hypoperfusion-induced lung dysplasia or restore the pulmonary function. Our study demonstrated that cell–cell communication and axon guidance, which are required for normal postnatal alveolar development, were partly damaged by pulmonary hypoperfusion. These results suggest that promoting cell–cell communication or supplementation with guidance molecules may repair lung dysplasia caused by neonatal pulmonary hypoperfusion, thereby suggesting new treatment options for pulmonary hypoperfusion-induced lung dysplasia.

Another important finding of the current study is that we found a clue that could be used to treat children with pulmonary hypoperfusion during the COVID-19 pandemic. COVID-19 causes persistent alveolar inflammation, which is controlled by axon guidance proteins [11, 25, 26]. Our results demonstrated that pulmonary hypoperfusion caused the loss of axon guidance (Fig. 6), which may explain the lack of patients with pulmonary hypoperfusion among the 17,065 pediatric CHD patients with severe COVID-19 accompanied with cardiovascular diseases [10, 27], suggesting that pulmonary hypoperfusion-induced loss of axon guidance protected children from COVID-19-induced immune storm. In addition, the COVID-19 virus infects cells with its spike protein, which can only play its role by binding to NRP1 [28]. In this way, NRP1 facilitates COVID-19 cell entry and infectivity [28]. Our data established that pulmonary hypoperfusion downregulated NRP1 and its receptors SEMA3a and SEMA7a (Fig. 8C–G), which may further explain why children with pulmonary hypoperfusion are resistant to COVID-19 infection. The transcriptomic profile of pulmonary hypoperfusion-influenced postnatal alveolar development may provide other important clues for CHD or PH treatment during COVID-19 pandemic, and requires further in-depth analysis.

An important limitation of the current study is that we did not evaluate the mechanism of disruption of cell communication and axon guidance by pulmonary hypoperfusion. We observed significantly higher cell apoptosis in the PAB group than in the sham group. Tempered cell apoptosis is required for the maturation of alveolar septa and parenchymal microvascular network, and cell apoptosis contributes to the thinning of the alveolar septa [28]. Apoptosis operates through molecular cascades, offering several potential signaling nodes that could intersect with axon guidance signaling pathways. For example, caspase-3 cleaves axon guidance molecules [29–31] Thus, it is possible that cell apoptosis, induced by pulmonary hypoperfusion, inhibits axon guidance signaling pathways (Fig. 9). This hypothesis needs to be verified in future research.

## Conclusion

In summary, the current study is the first to reveal the BP, CC, MF, and signaling pathways of postnatal alveolar development under the influence of pulmonary hypoperfusion. The results showed that pulmonary hypoperfusion damaged cell–cell communication and axon guidance, which provides clues for the treatment of pediatric CHD-associated lung dysplasia and COVID-19 infection. The underlying mechanisms by which pulmonary hypoperfusion disrupted cell–cell communication and axon guidance require further investigation.

## Supplementary Information

**Additional file 1: Tables S1–S3.** Primers, reagents, and antibodies.

## Data Availability

All of the data in the current study are available from the corresponding author upon reasonable request. The surgical video are accessiable online at https://doi.org/10.6084/m9.figshare.20296599. All RNA-seq data have been deposited in the GEO database (https://www.ncbi.nlm.nih.gov/geo; accession number GSE201522 and GSE206972). Information related to the primers, reagents, and antibodies is provided in Additional file 1: Tables S1–S3.

## Abbreviations

ALV: Alveolar stage
GO: Gene ontology
KEGG: Kyoto encyclopedia of genes and genomes
PA: Pulmonary artery
RNA-seq: RNA sequencing
H&E: Hematoxylin and eosin
FPKM: Fragments per kilobase of transcript sequence per million
TOF: Tetralogy of Fallot
PBS: Phosphate-buffered saline
DAPI: 4',6-Diamidino-2-phenylindole
AT2: Alveolar type 2
AT1: Alveolar type 1
VTI: Velocity–time integral
PPG: Peak pressure gradient
CHD: Congenital heart disease
