# Supplementary material for: Impaired cell–cell communication and axon guidance because of pulmonary hypoperfusion during postnatal alveolar development
Source: Respir Res. 2023 Jan 11;24:12. doi: 10.1186/s12931-023-02319-3 (PMC9833865; doi:10.1186/s12931-023-02319-3)
Supplement: Supplementary file 1 — Additional file 1: Tables S1–S3. Primers, reagents, and antibodies. [file 12931_2023_2319_MOESM1_ESM.docx]

Supplemental table S1 primer information

| Gene |  | Sequence (5'->3') |
| --- | --- | --- |
| Slit2 | Forward | ACCCAGGTAGCAGATTCTCTCTTCC |
|  | Reverse | GCAAAGACTAGGTGTCGGCGATG |
| Unc5c | Forward | CGGGGCGTGTTAAGTGTAGCAG |
|  | Reverse | GCCTCTGGAAATCTCCGGTTGTAC |
| Myl9 | Forward | GCCTAGCATGGTGTCCTGACTTG |
|  | Reverse | CGCAGCCTTCTTCATCCTTGAGA |
| Sema4a | Forward | GCCAATCCTGCCTCAGATGCTTC |
|  | Reverse | CCAAGACACTCAGAGCTGGCAAG |
| Sema3g | Forward | TCTATGTCAAACAAGCAGGGCATCC |
|  | Reverse | TGTCCAGAACTCCTCAGGCACTC |
| Plxna3 | Forward | AAAGCCCTCTGCCTATATCCCTCTC |
|  | Reverse | CACTGCAACACAAAGCCAAGACT |
| Sema7a | Forward | TCCCTCGGGCATCTCAGTTTAGAC |
|  | Reverse | GTTCAGTCCCTAGCGGCAGAAAG |
| Sema6a | Forward | CCTTCCTCTCACCTCCTCTTCTGTC |
|  | Reverse | TCCTTCGCAAGCCTTTGTCATTCC |
| Sema3b | Forward | GGTACACAGCCGTCTCCAGAGG |
|  | Reverse | GTCATTTCAGAGGGCACCAAGGG |
| Prkcz | Forward | TGGCTTCTTCCTTAGGTGTTGTGTG |
|  | Reverse | CAGCATTCCACTCCACTCCCTTTC |
| Mcm5 | Forward | TGGAGGCATGGAAAGAAAGCAGTG |
|  | Reverse | CAGCGGGACATCTCAGCAAACC |
| Mcm4 | Forward | CTTGTTTTCCAGCCCTCCTCAGATG |
|  | Reverse | TGCTTCATTCCCAGGTGTTTCAGTG |
| Ccna2 | Forward | CTTCTTTCCTTCCCTCCTTGCTGTC |
|  | Reverse | GGAGGCAGAGGTAGGTGGATCTC |
| Mcm6 | Forward | ACATCTGCCTCCTTCCATCTCTCG |
|  | Reverse | CTGCTGTGTAGTTGCTGCTAGGTG |
| Cdc20 | Forward | AATGGAGCAGCCTGGGGACTAC |
|  | Reverse | CACTGACCACGGAGCCACAATATAG |
| Mcm3 | Forward | GGTCCCCAATGCTCTCGTGATAAAG |
|  | Reverse | GTCCTCTGGTACGACATGCTTCTG |
| Cdk1 | Forward | CAGACGCTACCCAGGCTAAGAAAC |
|  | Reverse | GTCCACGCACCAAGACCTTCATC |
| Ccnb1 | Forward | CCTGAGCCTGAACCTGTTATGGAAG |
|  | Reverse | CCGTCGTGTGCATCAACATGAAATG |
| Ccnd2 | Forward | GAATGCTGAGAAGTGAGCCAGAAGG |
|  | Reverse | AACTAAAGCCTCCACAAACCCCAAG |
| Skp2 | Forward | CCAGAAGGCAGAGCGGATTGATG |
|  | Reverse | AATGGTTGGGAAATCAGGGAATGGG |

Supplemental table S2 Reagents

| Name | Company | Catalog No. |
| --- | --- | --- |
| Hematoxylin and eosin Kit | Beyotime biotech | C0105M |
| TUNEL Kit | Beyotime biotech | C1089 |
| HBSS | ThermoFisher Scientific, Pittsburgh, PA, USA | 14175103 |
| DNase | Worthington, Lakewood, NJ, USA | 9003-98-9 |
| RNase | Worthington, Lakewood, NJ, USA | 9001-99-4 |
| BCA kit | ThermoFisher Scientific | A53227 |
| paraformaldehyde(PFA) | Sigma-Aldrich | 158127 |
| 4',6-diamidino-2-phenylindole(DAPI) | ThermoFisher Scientific | D3571 |
| PureLink RNA Micro Scale Kit | Life Technologies, Carlsbad, California, USA | 12183016 |
| PrimeScriptTM reagent kit | Takara Bio, Kusatsu, Japan | RR037A |
| SYBR Green Power Premix Kits | Applied Biosystems, Foster City, California | 4368577 |
| RIPA lysis buffer | Beyotime, Shanghai, China | P0013B |
| NEB Next® UltraTM RNA Library Prep Kit | NEB, USA | E7760 |
| TruSeq PE Cluster Kit | Illumina | v3-cBot-HS |

| Supplemental Table S3 Antibodies | |  |
| --- | --- | --- |
| Name | Company | Catalog No. |
| CD31 | Abcam | ab281583 |
| Nrp1 | Abcam | ab81321 |
| Ki67 | Abcam | ab15580 |
| SEMA3a | Abcam | ab199475 |
| GAPDH | Abcam | ab8245 |
| Dylight 800- labeled affinity secondary antibodies | Kirkegaard & Perry Laboratories, Gaithersburg, MD | 072-07-15-06 |
